# The role of ABCB1 and ABCA1 in beta-amyloid clearance at the neurovascular unit in Alzheimer's disease

**DOI:** 10.3389/fphys.2013.00045

**Published:** 2013-03-13

**Authors:** Ayman ElAli, Serge Rivest

**Affiliations:** Laboratory of Neurosciences, Department of Molecular Medicine, Faculty of Medicine, CHU de Québec Research Center, Laval UniversityQuébec, QC, Canada

**Keywords:** Alzheimer's disease, blood-brain barrier, neurovascular unit, ABCB1, ABCA1, apolipoprotein E, beta-amyloid clearance

## Abstract

Alzheimer's disease (AD) is a progressive neurodegenerative disorder that affects elderly persons, evolving with age to reach severe cognitive impairment. Amyloid deposits and neurofibrillary tangles constitute the main pathological hallmarks of AD. Amyloid deposits are initiated by the excessive production and accumulation of beta-amyloid (Aβ) peptides in the brain. The dysfunction of the Neurovascular Unit (NVU) has been proposed to be causative in AD development, due to an impaired clearance of Aβ from the brain. Cells forming the NVU express several Adenosine Triphosphate ATP-Binding Cassette (ABC) transporters, among which ABCB1 and ABCA1 play an important role in Aβ processing. The drug transporter ABCB1 directly transports Aβ from the brain into the blood circulation, whereas the cholesterol transporter ABCA1 neutralizes Aβ aggregation capacity in an Apolipoprotein E (ApoE)-dependent manner, facilitating Aβ subsequent elimination from the brain. In the present minireview, we will summarize the contribution of ABCB1, and ABCA1 at the NVU in Aβ clearance. Moreover, we will outline and discuss the possible collaboration of ABCB1, and ABCA1 at the NVU in mediating an efficient clearance of Aβ from the brain.

## Introduction

Alzheimer's disease (AD) is a progressive neurodegenerative disorder that affects elderly persons. The pathogenesis of AD begins with mild memory deficits and evolves to reach total cognitive impairment and loss of executive functions (de Souza et al., 2009). It is now widely accepted that amyloid deposits and neurofibrillary tangles formation constitutes the core pathological hallmarks of AD (Selkoe, 2002). The sequential proteolytic cleavage of the Amyloid Precursor Protein (APP) produces beta-amyloid (Aβ) peptides, namely Aβ_1−40_ and Aβ_1−42_ (Hardy and Selkoe, 2002), which oligomerize to form small oligomers of 2–12 peptides (i.e., Aβ oligomers), and aggregate leading to Aβ plaques generation (Haass and Selkoe, 2007). Although the correlation between Aβ parenchymal deposition and cognitive decline still remains controversial, the detrimental role of soluble Aβ oligomers in the brain of AD patients (Lue et al., 1999) and in mouse models of AD (Cheng et al., 2007) has been demonstrated.

In more than 95% of AD cases, several environmental and genetic factors that influence Aβ processing have been reported to contribute in AD development (i.e., sporadic AD), among which Aβ clearance and elimination from the brain play a central role (Hardy and Selkoe, 2002). The remaining AD cases are caused by an excessive Aβ production in the brain due to mutations in either *APP* gene or the enzymes involved in its proteolytic cleavage (i.e., familial AD) (Levy-Lahad et al., 1995; Sherrington et al., 1995).

The levels of soluble Aβ oligomers in the brain play a crucial role in AD development, because their accumulation in brain parenchyma causes neuronal dysfunction that have been shown to take place even before the neurodegeneration cascade (Haass and Selkoe, 2007). In parallel, the early accumulation of Aβ oligomers in cerebral microvessels causes vascular dysfunction and contributes to the development of Cerebral Amyloid Angiopathy (CAA), which takes place in 80% of AD cases (Bell and Zlokovic, 2009). Interestingly, microvascular dysfunction has been reported at the early stages of AD pathogenesis (Zlokovic, 2005), outlining a central role of cerebrovascular dysfunction in AD development (Pimentel-Coelho and Rivest, 2012). Indeed, abnormalities at the Blood-Brain Barrier (BBB) have been reported in AD (Desai et al., 2007), supporting this hypothesis.

The BBB constitutes a physical barrier separating the peripheral circulation from the central nervous system (CNS), and plays a central and major role in controlling brain homeostasis and regulating brain microenvironment, by (1) precisely adjusting nutrient and oxygen delivery based on brain needs, (2) removing toxic metabolites from the brain into the blood, (3) protecting the brain from endogenous and exogenous toxic molecules, and (4) supporting parenchymal tissue viability (Löscher and Potschka, 2005). The BBB adopts a special phenotype characterized by a high transendothelial electrical resistance (TEER), thus preventing the free passage of blood-borne molecules and cells from brain entry (Zlokovic, 2008). Anatomically, the BBB is constituted by specialized endothelial cells that actively interact with Extracellular Matrix (ECM) proteins that form the perivascular space, pericytes, astrocytes, microglia and neurons, forming the neurovascular unit (NVU), which is the functional unit of the BBB (Zlokovic, 2011). The NVU has two functionally distinct sides, the luminal side facing the blood circulation, and the abluminal side facing the brain parenchyma (Hermann and ElAli, 2012). To fulfill its role in controlling brain homeostasis and regulating brain microenvironment, the cells forming the NVU are complemented by sophisticated active transport systems, such as ion channels, pumps, receptors, and transporters, among which are the transmembrane transporters belonging to Adenosine Triphosphate-Binding Cassette (ABC) transporter family.

The ABC transporter family consists essentially of 48 proteins in humans, which are subdivided into 7 sub-families (ABC1, MDR/TAP, MRP, ALD, OABP, GCN20, White) (de Lange, 2004; Leslie et al., 2005). Initially, ABC transporters were discovered by oncologists to be responsible for chemotherapy resistance (Biedler and Riehm, 1970). ABC transporters use energy generated from ATP hydrolysis to transport substrates across cell membranes (ElAli and Hermann, 2011), and have overlapping affinity for many lipophilic and amphipathic molecules, therefore physiologically considered as cell detoxification systems (de Lange, 2004). ABC transporters, depending on the sub-family, act either as gatekeepers by protecting organs from toxic compounds, or as transporters of bioactive molecules produced by cells (Leslie et al., 2005). Two ABC transporters have been shown to play important roles in AD pathogenesis; (1) ABC transporter sub-family B member 1 (ABCB1; i.e., Multi Drug Resistance Protein; Mdr-1) that acts as an efflux pump of xenobiotic molecules, and (2) the ABC transporter sub-family A member 1 (ABCA1; i.e., Cholesterol Efflux Regulatory Protein; CERP) that acts as an efflux pump for cholesterol and phospholipids from cell membranes to Apolipoprotein E (ApoE) and ApoA-I (Wahrle et al., 2005; Kuhnke et al., 2007).

Two main mechanisms govern Aβ clearance at the NVU, Aβ degradation by specialized enzymes, and Aβ transport across the BBB, from the brain into blood circulation (Nalivaeva et al., 2012; Sagare et al., 2012). Recently, it has been proposed that impaired Aβ transport, elimination, and clearance across the BBB/NVU constitute key steps in AD pathogenesis, as it causes an excessive accumulation of Aβ in brain parenchyma, inducing neuronal dysfunction and favoring Aβ plaques formation (Zlokovic, 2008). This hypothesis suggests that the dysfunction of the BBB/NVU causatively contributes to the pathogenesis of AD (Zlokovic, 2011), which is now under intense investigation. Several ABC transporters have been reported to be involved in Aβ processing, transport, and clearance at the NVU (Abuznait and Kaddoumi, 2012), among which ABCB1, and ABCA1 are the most studied and characterized. As such, in this minireview, we would like firstly to summarize the known roles of ABCB1 and ABCA1 expressed by the different cell types forming the NVU in Aβ clearance from brain parenchyma and microvessels into blood circulation, secondly to propose possible mechanisms for Aβ clearance through the NVU based on the existing literature, and finally to discuss the potential of ABCB1 and ABCA1 modulation as a new therapeutic strategy in CAA, and AD treatment.

## β-Amyoid clearance by brain endothelial cells

Being the first line of defense, brain endothelial cells express high level of ABC transporters, especially drug transporters that extrude toxic molecules from the brain, namely ABCB1. ABCB1 is highly expressed at the luminal side of brain endothelial cells, and is considered as marker of BBB maturity and functionality (Hermann and ElAli, 2012). It has been reported that soluble Aβ is transported across brain endothelial cells and eliminated from the brain into blood circulation, mainly via the low-density lipoprotein-related protein-1 (LRP-1) in collaboration with ABCB1 (Zlokovic, 2008). For instance, it has been shown that Aβ binds to ABCB1, and that the latter is directly involved in its active transport (Lam et al., 2001). In parallel, LRP-1 can bind directly Aβ or via ligands that bind Aβ, such as ApoE (Yamada et al., 2008). As such, LRP-1/ABCB1 seems to form an efficacious and complete transport system that coordinates Aβ transport and elimination from the brain. Interestingly, the pharmacological inhibition of ABCB1 decreased Aβ clearance *in vitro* (Lam et al., 2001; Kuhnke et al., 2007), outlining the direct involvement of ABCB1 in Aβ clearance. These *in vitro* observations were confirmed by *in vivo* studies where *abca1a/b*^−/−^ knockout mice intracerebrally injected with Aβ exhibited reduced clearance of these peptides compared to control littermates (Cirrito et al., 2005). In human, experiments on post-mortem tissue samples of AD patients showed decreased expression levels of ABCB1 in brain endothelial cells of microvessels that surround Aβ deposits (Vogelgesang et al., 2002), and almost no expression of ABCB1 in brain endothelial cells was detected in post-mortem tissue samples obtained from CAA patients (Vogelgesang et al., 2004).

It is noteworthy, that ABCB1 expression in brain endothelial cells decreases with physiological aging, as shown in long-lived B-N/F rats, which interestingly caused brain accumulation of exogenously administered Aβ (Silverberg et al., 2010). In human brain, ABCB1 expression has been reported to decrease in elderly subjects (Bartels et al., 2009). This observation is highly important, because the decreased expression of ABCB1 impeded NVU's capacity in acting as a gatekeeper for the brain, allowing endogenous and exogenous toxins, like Aβ, to accumulate in the brain, thus inducing neuronal dysfunction. Taken together, these reports and studies strongly suggest and advocate a central role of ABCB1 expression in brain endothelial cells in AD pathogenesis, by affecting Aβ transport across the brain endothelium.

**Figure 1:**
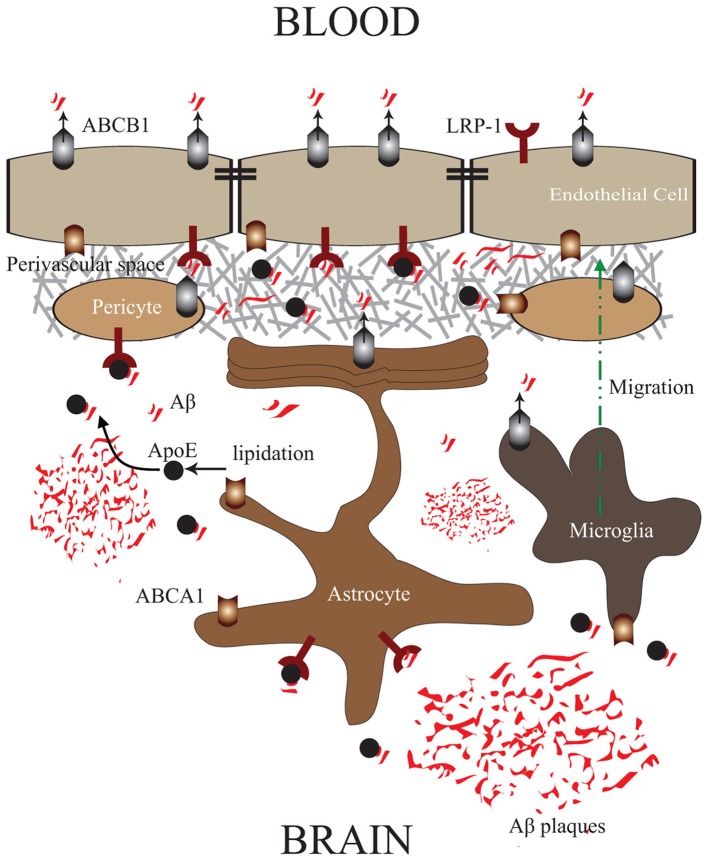
**ABCA1/ABCB1 Transport Systems in the NVU: Proposed mechanism via which ABCA1/ApoE—LRP-1/ABCB1 systems in the NVU cooperate in order to efficiently mediate Aβ clearance from brain parenchyma, and microvessels into blood circulation.** ABCA1 in brain endothelial cells, pericytes, astrocytes, and microglial cells enhances ApoE lipidation, and with the collaboration of ABCB1 facilitates Aβ trafficking into the perivascular space, where it can be subsequently eliminated into blood circulation via LRP-1/ABCB1 in brain endothelial cells.

In contrast to ABCB1, ABCA1 does not directly bind and extrude Aβ. In brain endothelial cells, ABCA1 is expressed at the abluminal side of the endothelium (Panzenboeck et al., 2002). Experiments conducted in *abca1*^−/−^ knock-out mice showed that ABCBA1 controls Aβ degradation fate and its ability to form aggregates, via an ApoE dependent manner, thus enhancing its clearance from the brain (Akanuma et al., 2008). Being a regulator of cholesterol efflux, ABCA1 controls protein levels of ApoE and its lipidation state, which constitutes an important factor in the capacity of ApoE to efficiently bind Aβ (Tokuda et al., 2000). As such, highly lipidated ApoE binds more efficiently Aβ and diminishes its capacity to aggregate by modulating its conformation (Wahrle et al., 2004; Holtzman et al., 2012). Interestingly, it was reported that PDAPP/*abca1* transgenic mice that overexpress ABCA1, have reduced Aβ deposits in brain parenchyma compared to their littermates, and almost complete absence of vascular Aβ deposits (Wahrle et al., 2008), outlining an efficient elimination of Aβ across the NVU. This observation is intriguing giving the fact that ABCA1 does not physically bind and eliminate Aβ. As such, we propose a mechanism via which the abluminal ABCA1, upon its activation, induces efficient ApoE lipidation, thus facilitating ApoE/Aβ interaction in the perivascular space, making Aβ more accessible to LRP-1/ABCB1 transport system at the NVU (Figure 1).

## β-Amyloid clearance by pericytes

Pericytes are tightly associated to the abluminal side of brain microvessels and covers approximately 25% of their circumference (Hellström et al., 2001). Brain pericytes act as vascular smooth cells (vSMC) by expressing several receptors of vasoactive molecules, therefore playing a major role in controlling brain microvascular tone (Armulik et al., 2011). Pericytes play an important role in regulating endothelial cell proliferation, survival, and migration (Hellström et al., 2001), and are required for BBB formation and induction by down-regulating genes associated with vascular permeability (Daneman et al., 2010), and inducing the functional activity of ABCB1 in brain endothelial cells (Al Ahmad et al., 2011). Moreover, some reports showed that brain pericytes behave like immune cells under stress by expressing the macrophage markers ED-2 and CD11b, and have some basal phagocytosis capacities (Kovac et al., 2011). Besides, it was reported that a high concentration of Aβ induces pericytes apoptosis (Verbeek et al., 1997), and differentially regulates pericytic genes at lower Aβ concentrations (Rensink et al., 2004). Interestingly, it has been shown that pericytes express LRP-1, and this expression has been shown to be modulated by Aβ loading (Wilhelmus et al., 2007). In parallel, ABCB1 has been reported to be expressed in pericytes (Berezowski et al., 2004; Bendayan et al., 2006). Taken together, these reports would suggest a potential role of the LRP-1/ABCB1 system in Aβ clearance, a hypothesis that merits further investigations. Recently, it has been reported that pericytes express ABCA1, which upon stimulation enhance ApoE lipidation without affecting the fate of the internalized Aβ (Saint-Pol et al., 2012). As such, it is very probable that the stimulation of ABCA1 in pericytes, in parallel to ABCA1 at the abluminal side of brain endothelial cells, further enhances ApoE production and lipidation at the perivascular space, diminishing the capacity of Aβ to aggregate in the perivascular space, and makes soluble Aβ more accessible to be cleared across the NVU (Figure 1).

## β-Amyloid clearance by astrocytes

Astrocyte endfeet cover up to 90% of brain microvessels, and play an essential role in inducing BBB features (Davson and Oldendorf, 1967). The presence of astrocytes is a prerequisite for BBB integrity by enhancing tight junction proteins expression and BBB functionality by inducing the expression of ABCB1 at the luminal side of brain endothelial cells (Abbott et al., 2006). Although the physiological level of ABCB1 expression in astrocytes is low and the exact role of this basal expression is totally unknown, it is well reported that under pathophysiological conditions, such as epilepsy, ABCB1 expression is highly increased and specifically at the endfeet of reactive astrocytes, thus playing a major role in epileptic drug resistance (Sisodiya et al., 2002). The expression levels of ABCB1 in astrocytes in AD are still to be investigated, but it would be highly surprising that its expression in astrocytes does not change in AD.

Nonetheless, it has been shown that astrocytes close to plaques express LRPs and internalize Aβ in an ApoE dependent manner (Arélin et al., 2002). The fate of Aβ uptaken by astrocytes is still elusive, as it has been proposed that the accumulation of Aβ can induce astrocytes dysfunction and contribute in the pathogenesis of AD (Nagele et al., 2003), or can be degraded (Wyss-Coray et al., 2003). Giving the fact that most intra-astrocytic Aβ are of neuronal origin (Nagele et al., 2003), and internalized mainly from cored plaques (Arélin et al., 2002), it is very probable, although speculative, that portions of internalized Aβ are trafficked toward endfeet facing the abluminal side of brain endothelial cells, where they can be eliminated in the perivascular space and be cleared into blood circulation by the brain endothelial cells transport system, LRP-1/ABCB1.

In addition, astrocytes express high level of ABCA1, which as discussed above, plays a crucial role in the lipidation of astrocyte-produced ApoE (Wahrle et al., 2004), thus facilitating Aβ clearance by reducing Aβ aggregation (Holtzman et al., 2012). As such, astrocytes contribute in Aβ clearance by making Aβ more diffusible and more accessible to be cleared across the NVU (Figure 1).

## β-Amyloid clearance by microglia

Microglial cells are the resident macrophages of the brain, constituting the first line of immune defense (Bellavance and Rivest, 2012). They act as continuous scavengers at the brain for Aβ plaques, debris, apoptotic neurons (Naert and Rivest, 2011), surveying closely the smallest changes in the brain, and exhibiting significant phagocytosis capacities (Naert and Rivest, 2011). Although microglial cells are closely associated to Aβ plaques *in vivo*, they do not appear to be efficient in clearing Aβ deposits (Bolmont et al., 2008). Interestingly, microglial cells are still able to internalize both fibrillar Aβ and soluble Aβ which would suggest that internalization of Aβ is not always accompanied by their degradation (Pan et al., 2011), outlining a different fate for these internalized Aβ peptides. Similar to astrocytes, ABCB1 expression in microglia is low under physiological conditions (Wolf et al., 2012), but has been shown to be increased in epilepsy (Löscher and Potschka, 2005). The expression levels of ABCB1 in microglia cells in AD are totally unknown, but as microglial cells have the ability to internalize Aβ, it would be probable that ABCB1 in microglial cells plays a role in Aβ transport. Similar to astrocytes, microglial cells express high levels of ABCA1 and contribute to ApoE production and lipidation in the brain, therefore contribute to Aβ clearance (Hirsch-Reinshagen et al., 2004) (Figure 1).

## Pharmacological modulation of ABCB1 and ABCA1 at the level of NVU as a therapeutic approach

ABCB1 plays a key role in Aβ transport and clearance at different levels of the NVU. This role could be highly relevant in developing new strategies to treat AD. Indeed, ABCB1 is induced following activation of the Orphan Nuclear Receptor (ONR), Pregnane X Receptor (PXR) in brain microvessels of humans (Bauer et al., 2006). A clinical study conducted in AD patients with mild cognitive deficits concluded that both doxycycline and rifampicin efficaciously reduce the cognitive decline in treated patients (Loeb et al., 2004), possibly and partly via a mechanism involving ABCB1, as both molecules have been reported to induce ABCB1 expression (Wolf et al., 2012). In parallel, it has been shown, in a mouse model of AD that inducing the expression of ABCB1 by stimulating PXR, highly decreased Aβ brain accumulation (Hartz et al., 2010). More recently, it has been reported that the stimulation of Retinoid X Receptor (RXR) using the agonist bexarotene in a mouse model of AD enhanced soluble Aβ clearance in an ApoE dependent manner (Cramer et al., 2012). It is noteworthy that RXR binds as a heterodimer with PXR to the PXR-responsive element regulating *abcb1* gene expression (Geick et al., 2001). Finally, ABCB1 constitutes a main target gene of nuclear receptors, as even the stimulation of Liver X Receptor (LXR) with agonists has been reported to potently induce ABCB1 expression in brain microvessels (ElAli and Hermann, 2012). The induction of ABCB1 by nuclear receptors' stimulation could be a highly attractive strategy, because it allows in parallel to induce the expression of ABCA1 and ApoE, and the lipidation of the latter (Koldamova et al., 2003; Donkin et al., 2010).

## Conclusion

The expression of ABCB1 and ABCA1 in all cells forming the NVU is of high importance, because it would constitute an elegant mechanism via which the ABCA1/ApoE and LRP-1/ABCB1 systems work in concert and cooperate to facilitate Aβ transport from plaques towards brain microvessels, and its subsequent clearance into blood circulation. Most of the parenchymal deposits are relatively far from functional brain microvessels, as such the mechanism proposed here suggests Aβ trafficking towards the perivascular space and abluminal side of brain endothelial cells, in order to be cleared into blood circulation. As such, we believe that the up-regulation and function of ABCB1 and ABCA1, not only in brain endothelia, but in all cells forming the NVU (Figure 1), may enhance Aβ clearance from the brain more efficiently, consequently reducing Aβ deposition in both parenchyma and perivascular space. This mechanism provides a novel approach to be considered in parallel with other strategies in treating CAA and AD.

### Conflict of interest statement

The authors declare that the research was conducted in the absence of any commercial or financial relationships that could be construed as a potential conflict of interest.
